# Necrolytic migratory erythema following prolonged continuous subcutaneous dasiglucagon administration: a rare dermatologic adverse event

**DOI:** 10.1530/EDM-25-0006

**Published:** 2025-04-29

**Authors:** Lucas Weschle, Jenny Potratz, Frank Rutsch, Alexander Humberg, Sandra Oesingmann, Andreas Pascher, Alexander S Busch, Katja Masjosthusmann

**Affiliations:** ^1^ Department of General Pediatrics, University of Münster, Münster, Germany; ^2^ Department of General, Visceral and Transplant Surgery, University of Münster, Münster, Germany

**Keywords:** necrolytic migratory erythema, dasiglucagon, congenital hyperinsulinism, neonatal hypoglycemia

## Abstract

**Summary:**

Clinical management of congenital hyperinsulinism (CHI) remains a significant challenge due to its complex pathophysiology and the limitations of available therapies. Dasiglucagon, a synthetic glucagon analog, represents a novel approach to managing CHI, particularly in patients where conventional therapies fail. This report discusses a rare case of prolonged continuous subcutaneous dasiglucagon use in a neonate with CHI. Despite initial stabilization of glycemic levels, the patient developed necrolytic migratory erythema (NME), a rare dermatological condition associated with hyperglucagonemia, during dasiglucagon therapy. The patient further experienced severe malnutrition, zinc and amino acid deficiencies, and sepsis. Following the discontinuation of dasiglucagon therapy due to these severe side effects, the patient’s skin and nutritional status improved markedly. However, glycemic control required subtotal pancreatectomy. This report underscores the potential of dasiglucagon in CHI management but highlights the importance of close monitoring during prolonged therapy.

**Learning points:**

- NME, a rare but severe condition, appears to be associated with prolonged continuous subcutaneous dasiglucagon therapy, requiring early recognition and intervention.
- Close monitoring is essential during prolonged continuous subcutaneous dasiglucagon therapy to detect potential adverse effects, focusing on dermatological conditions, nutrient deficiencies or signs of infection.
- Multidisciplinary care is crucial to manage CHI with dasiglucagon, ensuring a comprehensive approach that addresses both glycemic control and potential side effects.

## Introduction

Dasiglucagon, a synthetic glucagon analog, represents a promising candidate for the treatment of severe hypoglycemia in patients with congenital hyperinsulinism (CHI) (1). In contrast to glucagon, its stability in aqueous solutions allows for ready-to-use formulations and continuous subcutaneous infusion, simplifying management and reducing the risk of rebound hypoglycemia. Dasiglucagon activates hepatic glucagon receptors, thereby stimulating glycogen breakdown, glucose release from the liver and gluconeogenesis, thus increasing blood glucose levels. Pharmacodynamic studies have demonstrated a rapid increase in blood glucose following subcutaneous dasiglucagon administration, similar to native glucagon (2). Current clinical trials are exploring its use in patients with CHI. A recent phase 3 trial indicated that dasiglucagon, particularly when administered via continuous subcutaneous infusion, may help improve glycemic stability in patients with CHI (1).

In this case report, we describe a severe adverse event associated with prolonged continuous subcutaneous dasiglucagon therapy: necrolytic migratory erythema (NME). NME is a rare dermatological condition most commonly linked to prolonged exposure to elevated glucagon levels, as seen in rare pancreatic neuroendocrine tumors such as glucagonomas. The characteristic rash presents as bright red, scaly lesions with superficial epidermal necrosis, typically spreading in a centrifugal pattern and predominantly affecting areas subject to higher friction (3). While suspected cases of NME associated with dasiglucagon have been reported previously, this case provides the first detailed characterization of this potential adverse effect during prolonged continuous therapy.

## Case presentation

**Figure 1:**
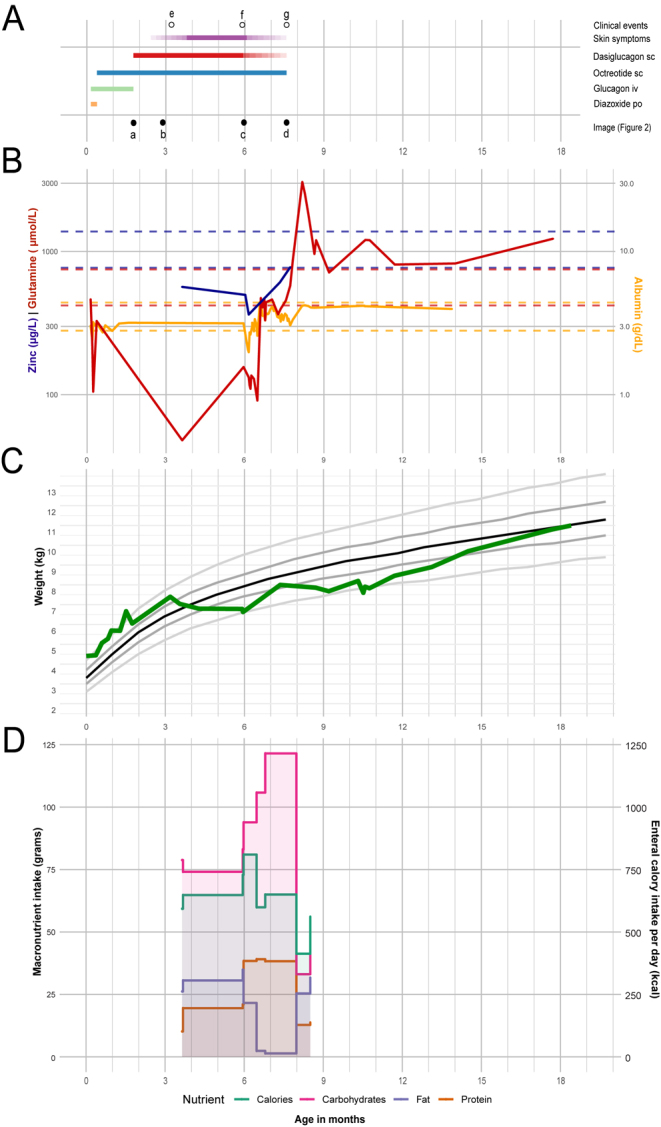
Clinical timeline, biomarker serum levels, weight by age and enteral nutritional status information. (A) Dots mark clinical events (empty) or refer to images (filled) in Fig. 2. Colored bars indicate periods. The x-axis denotes time in months. Following a diazoxide trial (orange bar), therapy with intravenous glucagon (green bar) and subcutaneous octreotide (blue bar) was initiated. In our observation, skin involvement (purple bar) was initially documented (e) a few weeks after the initiation of subcutaneous dasiglucagon (red bar), while the parents took notice of skin changes before this. Subcutaneous dasiglucagon was tapered (fading color) following sepsis (f). This was paralleled by a gradual improvement in the skin affection. Ultimately, a subtotal pancreatectomy was performed (g). (B) Serum zinc (blue), glutamine (red) and albumin (yellow) trajectories over time. Solid lines connect individual measurements, while dashed lines indicate reference ranges. Glutamine measurements are shown as an example of general amino acid levels. (C) Patient weight trajectory over time (green line), with the solid black line indicating the 50th percentile and solid gray lines representing the 5th, 25th, 75th and 95th percentiles of the reference population. (D) Enteral macronutrient intake and enteral calories per day during dasiglucagon treatment.

A male neonate presented with severe hypoglycemia, with a blood glucose level of 0.4 mmol/L shortly after birth. The boy had been born at a gestational age of 36 weeks and 4 days with a birthweight of 4,410 g. Initial management of suspected CHI included high-dose intravenous glucose. Five days after birth, he was transferred to our university hospital. Conservative management with a maximum of 35 g/kg/d glucose was continued. A trial of diazoxide was ineffective. Subsequently, intravenous glucagon infusion at 20 μg/kg/h (2 mg/day) and subcutaneous octreotide therapy 20–40 μg/kg/d were initiated (Fig. 1A), stabilizing blood glucose levels and reducing intravenous glucose dependency. The patient experienced complications, including mild hypertrophic non-obstructive cardiomyopathy due to underlying CHI, central venous catheter-associated thrombosis due to intravenous glucose or glucagon dependency and mild compensated metabolic acidosis associated with glucagon therapy. At this early stage of intravenous glucagon therapy, his skin showed no noticeable signs of affection despite a family history of atopic dermatitis and the potential risk of skin reactions associated with the treatment.

## Investigation

Genetic testing confirmed compound-heterozygous mutations in the *ABCC8* gene, specifically c.2506C>T p.(Arg836*) and c.2992C>T p.(Arg998*), both classified as pathogenic (class 5). Segregation analysis revealed that the c.2506C>T mutation was maternally inherited, while the c.2992C>T mutation was inherited from the father. The genetic findings resulted in the diagnosis of CHI due to compound-heterozygous *ABCC8* mutations.

## Treatment

**Figure 2:**
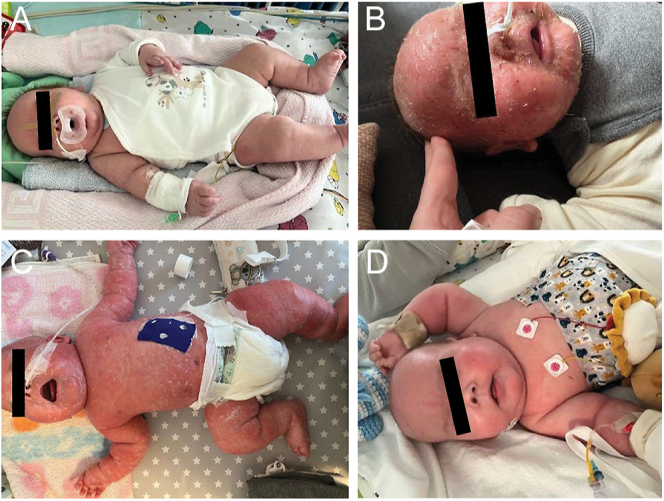
Clinical images. Clinical images refer to timepoints depicted in Fig. 1 and represent the clinical presentation before dasiglucagon treatment (A), during treatment (B), shortly before admission to the PICU (C) and shortly after subtotal pancreatectomy (D).

Over the course of almost 8 weeks, the patient could not be fully weaned off intravenous glucagon due to insufficient glycemic control. Genetics confirmed diazoxide resistance and octreotide alone (maximum dose 40 mg/kg/d) remained insufficient for glucose control. This led us to transfer the patient, at 8 weeks of age, to a specialized hospital with the option of continuous subcutaneous dasiglucagon therapy via an insulin pump. At the time of transfer, the patient was clinically stable and on full enteral feeds. His skin showed no abnormalities (Fig. 2A), and his enteral glucose intake was 10 g/kg/d. Three weeks later, the patient was discharged and attended routine follow-up care in our outpatient clinic while continuing subcutaneous dasiglucagon therapy via insulin pump (40–60 μg/h) with octreotide maintained at 4 × 6 μg subcutaneously. In addition, he received care in the outpatient clinic of the specialized hospital to ensure comprehensive monitoring of the dasiglucagon therapy. He was monitored with a continuous glucose monitoring sensor. He also received a daily enteral protein supplement enriched with essential nutrients, starting at 16 weeks of age, in response to gradually developing hypoproteinemia and amino acid deficiency.

During a regular outpatient clinic visit for continuous subcutaneous dasiglucagon therapy monitoring, 4 months after start, hypernatremia was diagnosed and the patient was transferred to our hospital for treatment. At the time of transfer, the dasiglucagon dose per day was reduced to 20–30 μg/h. Upon admission, the patient presented with erythematous and swollen skin, along with intermittent erythematous papules on his back. Retrospectively, both the parents and our medical team had observed skin changes following the initiation of subcutaneous dasiglucagon therapy (Fig. 2B). However, during a regular outpatient clinic visit, we documented the first clear occurrence of skin changes at 97 days of age, i.e. 43 days after the start of continuous subcutaneous dasiglucagon therapy.

The initiation of dasiglucagon therapy was followed by a downward crossing of multiple weight percentile lines in serial measurements, reflecting a consecutive weight loss of 770 grams (Fig. 1C). Amino acid, albumin and zinc levels were significantly reduced, with values falling below age-specific reference ranges (Fig. 1B). There was no history of excessive vomiting or diarrhea. Based on the patient’s history and clinical presentation, we strongly suspected NME. Given the patient’s severe skin condition, a central line was chosen to ensure secure intravenous access and targeted intravenous substitution of zinc, trace elements, amino acids and fatty acids was initiated to treat malnutrition.

While we initiated further diagnostic workup for NME together with dermatology (Fig. 2C), the patient was admitted to our pediatric intensive care unit (PICU) due to Staphylococcus aureus-associated central line sepsis. Due to the severity of the patient’s condition, we refrained from performing a skin biopsy for histological analysis. We reduced the dasiglucagon dose to 18–27 μg/h, increased the octreotide dosage to 40 μg/d subcutaneously and supplemented with increased oral glucose intake. Dasiglucagon treatment was tapered and ultimately discontinued 4 weeks after admission to the PICU. The entire treatment totaled 6 months. During the hospital stay, the patient exhibited a persistent susceptibility to infections, attributed to his compromised skin integrity and zinc deficiency, which also led to recurrent complications with the central lines. Despite high doses of octreotide and a glucose intake of 17 g/kg, stable blood glucose levels could not be achieved. Consequently, a subtotal pancreatectomy was performed, with approximately 97% of the pancreas removed. As the patient had developed a feeding disorder, largely refusing oral intake, a PEG tube was placed to ensure adequate nutritional support postoperatively.

## Outcome and follow-up

As anticipated, the patient developed diabetes mellitus postoperatively and continuous subcutaneous insulin infusion was initiated. After the discontinuation of dasiglucagon and the definitive treatment with subtotal pancreatectomy, the patient’s condition improved significantly. Enzyme replacement therapy was initiated to manage exocrine pancreatic insufficiency, ensuring effective digestion and nutrient absorption. His skin issues resolved almost entirely (Fig. 2D), and he began thriving, successfully reaching his developmental milestones. The PEG tube was subsequently removed at 17 months of age following significant improvement in the patient’s feeding abilities.

## Discussion

Dasiglucagon has shown promise in managing severe hypoglycemia through its rapid glucose-raising effects. While short-term use demonstrates efficacy and tolerability in a growing number of therapeutic indications (4), long-term treatment may be associated with risks of adverse effects similar to those observed with prolonged glucagon exposure (5), as both substances share mechanisms of glucagon receptor agonism. We report the case of an infant with CHI who developed severe NME during prolonged continuous dasiglucagon treatment, requiring its discontinuation.

The pathogenesis of NME is complex and not fully understood. This rare condition has an estimated incidence of approximately 1 in 20 million cases per year and can manifest outside the context of classical glucagonoma syndrome (6). However, hyperglucagonemia is the cause of profound changes in metabolism such as inducing a catabolic state that disrupts essential nutrients, including zinc, amino acids and fatty acids. These nutrient deficiencies, classified within the broader family of deficiency dermatoses, impair epidermal function and trigger inflammatory processes that lead to the characteristic skin fragility and ulceration. This multifactorial malnutrition model proposes that glucagon-induced nutrient deficiencies destabilize epidermal integrity, predisposing to cellular breakdown, inflammation and impaired wound healing (6). Zinc, particularly, plays a key role in protein and lipid metabolism, with deficiency potentially contributing to impaired skin repair (3). Hypoaminoacidemia – due to glucagon-driven gluconeogenesis – limits amino acid availability, reducing peptide synthesis and increasing inflammatory mediator production in the epidermis. In this context, NME has been previously described in congenital glutamine synthase deficiency (7). Essential fatty acid deficiency, compounded by hypoalbuminemia, may exacerbate inflammation through arachidonic acid pathway dysregulation (8). This inflammatory environment not only compromises skin repair but may also predispose lesions of NME to secondary complications, such as superimposed infections, which are most commonly caused by *Staphylococcus aureus* and *Candida albicans* (9). Supplementation of zinc, amino acids and fatty acids has been shown to improve skin symptoms, underscoring the importance of addressing these deficiencies in treatment strategies for NME (10).

Our case underscores several key lessons for clinicians managing patients with CHI. First, it highlights the importance of rigorous monitoring during prolonged continuous treatment with synthetic glucagon analogs to detect early signs of potential adverse effects such as dermatological conditions or nutrient deficiencies, including regular weight controls. Clinicians should remain vigilant for symptoms indicative of NME, such as erythematous skin lesions or systemic signs of malnutrition. In addition, the case demonstrates the value of integrating targeted nutritional support, including supplementation with zinc, amino acids and essential fatty acids, to mitigate the risk of complications associated with prolonged glucagon receptor agonism. Regular assessments of nutrient levels and early interventions may play a critical role in preventing adverse outcomes. Finally, this case emphasizes the need for a multidisciplinary approach involving endocrinologists, diabetologists, dermatologists, surgeons, metabolic specialists and nutritionists to ensure comprehensive care. By combining pharmacological treatment with proactive management of potential side effects, including the timely consideration of surgical options, clinicians may optimize patient outcomes while minimizing potential risks associated with long-term therapy of this otherwise promising glucagon analog, dasiglucagon.

In our case, the maximum administered dose of dasiglucagon was 60 μg/h. In a recent study, the highest reported infusion rate was 70 μg/h, with some patients up-titrated to this dose (1). Skin-related adverse events were more frequent in the dasiglucagon-treated group. However, the precise threshold at which erythema develops remains unclear.

This case report is limited by its single-patient focus and the lack of comparative data, making it difficult to determine whether the observed complications are generalizable to other patients receiving prolonged continuous dasiglucagon therapy. Furthermore, we cannot exclude that co-existing factors such as comorbidities or concurrent medications may have contributed to the observed adverse effects.

In conclusion, this case report sheds light on the potential adverse effects of prolonged continuous dasiglucagon therapy, particularly its association with NME and systemic complications such as severe malnutrition, amino acid and zinc deficiencies and increased susceptibility to infections. However, this report does not establish a direct causal link between dasiglucagon and NME. While dasiglucagon remains a promising treatment of CHI, this case emphasizes the critical importance of close monitoring during prolonged continuous therapy to identify early signs of potential dermatological adverse events and nutrient deficiencies.

## Declaration of interest

The authors declare that there is no conflict of interest that could be perceived as prejudicing the impartiality of the work reported.

## Funding

This research did not receive any specific grant from any funding agency in the public, commercial or not-for-profit sector.

## Patient consent

Written informed consent for publication of the clinical details and clinical images was obtained from the parents of the patient.

## Author contribution statement

All authors made individual contributions to authorship. LW, JP, FR, AH, SO, AP, ASB and KM were involved in the diagnosis and/or management of this patient. AP was responsible for the surgical management of this patient. All authors reviewed and approved the final draft.
